# THE SWEDISH NEEDS ASSESSMENT PROJECT TO ENHANCE LIFE FOR PEOPLE LIVING WITH SPINAL CORD INJURY: SYNTHESIZED EVIDENCE OF NEEDS TO BE MET, RESEARCHER PRIORITIES, AND KNOWLEDGE GAPS

**DOI:** 10.2340/jrm-cc.v8.43069

**Published:** 2025-08-05

**Authors:** Jeanette MELIN, Emelie AXWALTER, Gunilla ÅHRÉN, Katharina Stibrant SUNNERHAGEN, Åsa LUNDGREN NILSSON, Johanna WANGDELL

**Affiliations:** 1 Gothenburg Competence Centre for Spinal Cord Injury, University of Gothenburg and Sahlgrenska University Hospital, Gothenburg, Sweden; 2 Department of Leadership, Demand and Control, Swedish Defence University, Karlstad, Sweden; 3 School of Social Sciences, Södertörn University, Stockholm, Sweden; 4 Department of Health and Caring Sciences, Linneaus University, Sweden; 5 Department of Clinical Neuroscience, Sahlgrenska Academy, Institute of Neuroscience and Physiology, Gothenburg, Sweden; 6 Department of Neurocare, Sahlgrenska University Hospital, Gothenburg, Sweden; 7 Department of Hand Surgery, Institute of Clinical Sciences, University of Gothenburg, Gothenburg, Sweden; 8 Centre for Advanced Reconstruction of Extremities, Sahlgrenska University Hospital/Mölndal, Gothenburg, Sweden; 9 Department of Occupational therapy and Physiotherapy, Sahlgrenska University Hospital, Gothenburg, Sweden

**Keywords:** disability, human rights, review, social security, spinal cord injury, quality of life

## Abstract

**Objective:**

The aim of this report is to describe the aggregated insights and key findings from a Swedish need assessment project. The project comprised 3 parallel studies identifying [a] needs to be met, [b] research questions, and [c] knowledge translation gaps related to enhancing the lives of people with spinal cord injury (SCI).

**Methods:**

The project included people living with SCI, their relatives, health professionals, and personal care assistants. Analyses were conducted with the aim of identifying commonalities and connections between the results of the 3 individual studies.

**Results:**

The aggregated insights and key findings from this project can be summarized into 3 themes. First, a well-functioning healthcare, rehabilitation, and supporting system must have a holistic perspective on what it means to live with SCI. Second, aging is about living with the SCI and getting old with the SCI. Third, when family members receive their own support, they can provide good support to the person living with SCI.

**Conclusion:**

New evidence from the Swedish needs assessment project reveals the needs that currently have the greatest impact on improving the lives of people with SCI, which can guide researchers, healthcare providers, and knowledge translators.

## LAY ABSTRACT

This paper reports aggregated insights and key findings from a Swedish needs assessment project. The project has addressed [a] needs to be met, [b] research questions, and [c] knowledge translation gaps related to enhancing the lives of people with spinal cord injury (SCI). The results emphasized that people living with SCI have basic needs regarding relationships, being loved, and feeling secure – similar to everyone else – however, aspects related to the injury add to and/or challenge these basic needs. SCI impacts most parts of life and requires greater engagement with healthcare and authorities. Due to the complexity of living with SCI, understanding and flexibility regarding individual needs are desirable for an enhanced life.

Spinal cord injury (SCI) affects many individuals worldwide and often leads to lifelong disabilities. While the magnitude and repercussions of the injury vary greatly among individuals, it is well-established that beyond surviving SCI, good quality of life is also important. Thus, the care and rehabilitation must focus on how to best provide support to enhance the lives of people living with SCI (1–3).

The United Nations Universal Declaration of Human Rights states that everyone has the right to life, liberty, work, education, leisure, and societal participation (4), and that persons living with disabilities should have equitable lives compared to others (5). However, persons living with different disabilities face unique challenges to enjoying an enhanced life. For instance, people living with SCI often have some degree of physical impairment, a sense of loss, and complications (e.g. neuropathic pain and spasticity) that necessitate specific considerations when participating in social activities, relationships, and occupation and leisure activities (2, 3). Moreover, living with SCI is not a static but rather a dynamic state that includes continual psychosocial challenges over time (6), which can affect needs for an enhanced life. Therefore, it is essential to investigate the issues that matter most to persons living with a specific condition, to ensure focus on the most important questions in research, innovation, and development activities, and the implementation thereof.

While care and rehabilitation play critical roles in supporting enhanced lives of people with SCI, other sectors of society must also offer support and adjustments – for example personal care assistance (c.f., 7, 8) as well as accessible environments at home, at work, in the community, and in the natural environment (c.f., 9, 10). There remains a need for more comprehensive understanding and knowledge regarding the specific needs and factors that contribute to an enhanced life for people living with SCI. For this purpose, the Swedish need assessment project was initiated in 2021. In order to bring light over this multifactorial theme, the project comprised 3 parallel studies to identify [a] needs to be met (11), [b] research questions (12), and [c] knowledge translation gaps (13) related to an enhanced life for people living with SCI. In the present paper, we aim to report the aggregated insights and key findings from the project. By bringing together the different components of the project – which may not be apparent when each study is viewed in isolation – this paper highlights overarching patterns that are readily accessible to guide researchers, healthcare providers, and knowledge translators. It offers up-to-date insights into the issues that matter most for improving the lives of people living with SCI.

## MATERIALS AND RESULTS

**Fig. 1:**
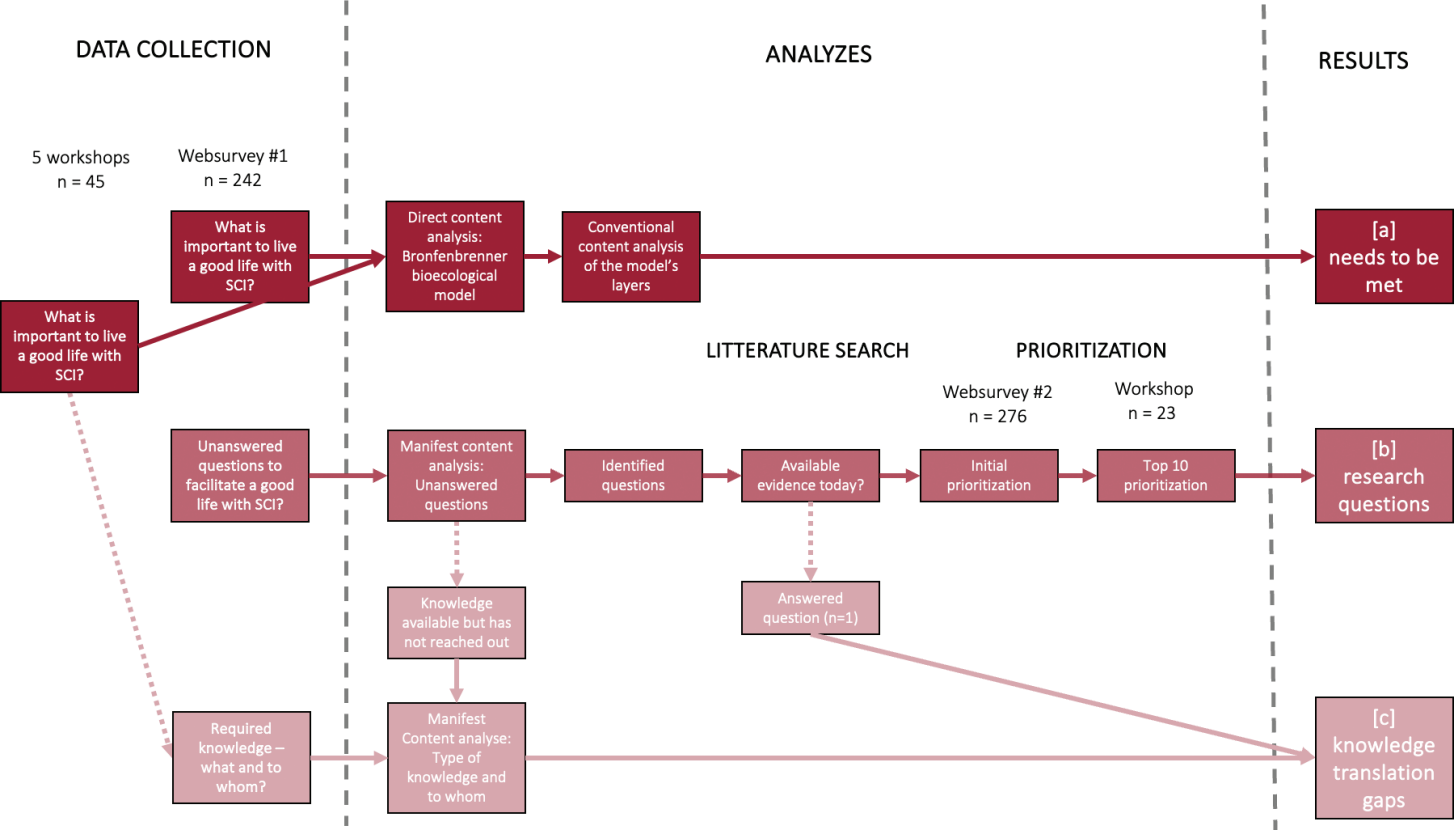
Illustration of data collection, analyses, and results for the 3 parallel studies in the Swedish needs assessment project. SCI: spinal cord injury.

**Fig. 2:**
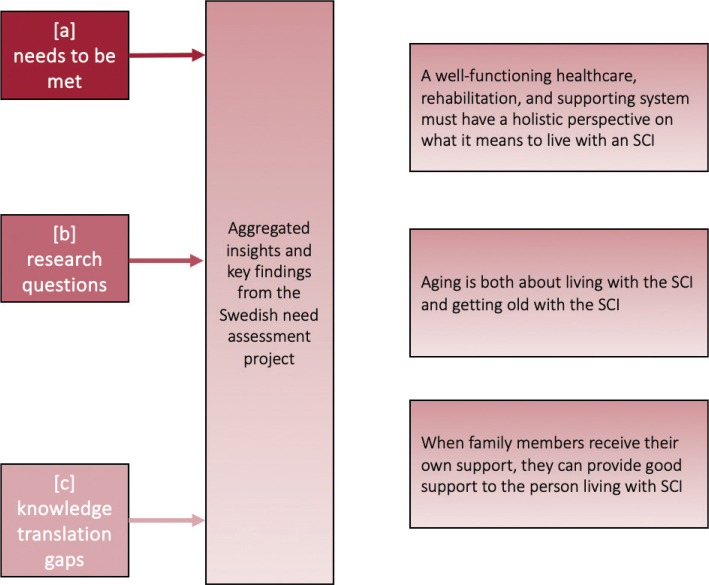
Illustration the aggregated insights and key findings from the Swedish need assessment project.

The Swedish need assessment project encompassed 3 cross-sectional studies (11–13) that combined workshops and surveys including people living with SCI and their relatives, as well as health professionals and personal care assistants working with people with SCI. The results were analyzed to generate data to respond to the aim of each study (Table I). Fig. 1 presents the process of data collection, and additional details regarding the participants, methods, and results are reported in the original studies. In the present analysis, we derived aggregated insights and key findings from the project, with the aim of finding commonalities and connections between the results of the individual studies. More specifically, the key findings from each study were placed side by side, and through discussions within the research team, links were identified between overlapping elements. These connections were then synthesized into higher-order themes (Fig. 2) through an iterative process, moving back and forth between the overarching themes and the original study findings. The synthesis thus followed a process similar to a content analysis. The research team conducting the analyses consisted of 2 researchers, each with more than 10 years of experience in both qualitative and quantitative SCI research (one of whom also has more than 30 years of clinical experiences) along with a language specialist and an individual living with SCI.

**Table I:** Aims of each of the 3 parallel studies in the Swedish needs assessment project

Study	Aim
[a] Wangdell et al. (11)	Identify needs contributing to an enhanced life for people living with spinal cord injury in Sweden
[b] Melin et al. (12)	Identify the top ten research priorities identified through the priority-setting partnership
[c] Melin et al. (13)	Present knowledge translation gaps that must be bridged to enhance life for people with spinal cord injury

Overall, people living with SCI have basic needs regarding relationships, being loved, and feeling secure – similar to everyone else – however, aspects related to the injury add to and/or challenge these basic needs (study [a]). Such aspects can be categorized as relating to “Physical health” and “Body functions” (study [b]). For example, people living with SCI who are suffering from pain and secondary complications are likely to have difficulties appreciating their social life, relationships, leisure activities, etc. Living with SCI also means that interactions with healthcare and societal support must become a large aspect of life, encroaching on time usually dedicated to relations, work, and leisure (the microsystem in study [a]). Moreover, the most commonly mentioned knowledge gap was the complexity of incorporating all aspects related to life with SCI (study [c]).

### A well-functioning healthcare, rehabilitation, and supporting system must have a holistic perspective on what it means to live with an SCI

Study [a] revealed that people with SCI are willing to take ownership of their lives. However, the injury and its consequences mean that this must be a different kind of ownership compared to others and necessitates specific interactions with healthcare and societal support structures and authorities. The surrounding systems (e.g. government) set “the rules” and create prerequisites for meeting basic needs for an enhanced life for people living with SCI. Moreover, given the regional organization of healthcare services in Sweden, it is essential that the surrounding system actively works to overcome inequalities – such as differences in access to support and care – based on where a person lives to ensuring equal opportunities regardless of geographic location.

Study [b] elucidates an optimal set-up of this organization, reflected by the research question categories “Care and rehabilitation” and “Community support and personal care assistance”. Specifically, priority #1 was the research question “What kinds of specialist care, from the emergency stage to rehabilitation and lifelong follow-up, should be available?”. Promising research has been conducted with different targeted interventions throughout the chain of care (see summary in study [a] and initial evidence in Study [b]’s Appendix 1). However, a holistic view of efficient long-term rehabilitation still needs to be further developed, implemented, and evaluated throughout different health care systems.

Study [c] demonstrated that healthcare professionals beyond the SCI units (e.g. primary care, emergency departments, and municipality care) were particularly in need of knowledge related to SCI. This was also reflected in priority #4 of study [b], addressing how non-specialist institutions can best respond to people with SCI. With regard to knowledge translation gaps, study [c] also emphasized that authorities needed more knowledge – for example, about the complexity of living with SCI but also in terms of body functions and environmental factors. Furthermore, the importance of positive attitudes, both towards oneself and from healthcare and authorities, was a theme cutting throughout all systems in study [a], and has the potential for improving a holistic and individual understanding throughout the lifespan.

### Aging is both about living with the SCI and getting old with the SCI

In the initial phase of study [b], up to 27% of identified issues concerned aging with SCI. However, none of the top ten priorities specifically addressed aging. This is most likely because these issues were often vaguely defined and thus collapsed into 1 broad research question (priority #12). It was also addressed as part of changes over time for incomplete- and non-traumatic injuries or women’s health. Nevertheless, given the significance of aging with SCI in the initial phase of study [b], and the need for flexibility throughout the lifespan highlighted in study [a], we interpret aging with SCI as an overarching component affecting an enhanced life for persons with SCI. As emphasized in study [a], we must consider changes throughout the entire life – for example, receiving help with secondary complications, following up on injury-related aspects, and obtaining support regarding “normal” life changes and life events, such as aging, changes in family constellations, and work situations.

Furthermore, as discussed in study [a], the Swedish supporting system after the retirement age could produce inequalities and may have negative repercussions regarding an enhanced life for older people living with an SCI. Specifically, this population will no longer have the same rights, because the Swedish Act concerning support and service for people with certain functional impairments (LSS) only applies to persons below retirement age. Accordingly, in study [b], priority #9 was “What are the implications of this restriction for people with SCI when they turn 65, and for those who get SCI in old age?”.

### When family members receive their own support, they can provide good support to the person living with SCI

A person’s SCI will inevitably affect their family and close ones, and these affected individuals may require their own support for coping with and adjusting to the trauma. In study [b], priority #8 was how to provide support to family members. This should be examined together with one of the key points from study [a], emphasizing that healthcare must better engage – rather than push away – family members and close ones. Failing to support family members may have double negative consequences. Firstly, they may feel even worse when they see their loved ones facing difficult times and cannot help. Secondly, if family and close ones are not getting sufficient support to handle their situation, they may not be able to support the person living with the SCI. In study [b], priority #5 was evidence for efficient support and treatment to cope with the new and ever-changing life situation, demonstrating the importance of support, in which relatives and also peer supporters will likely play an important role. Further, 29% of the initial questions that needed answers (study [b]) were related to mental health and relationships.

## DISCUSSION

People with SCI have many needs that are the same as other persons in general, as well as shared among other people living with SCI. However, living with SCI can entail several additional challenges. Moreover, each person living with SCI has a unique injury and context of living, such that the requirements for an enhanced life are highly individual. In each of the 3 studies, as well as in the key findings and aggregated insights presented in this report, it is clear that for people living with SCI in Sweden it is a complex situation, and that human rights statements are not fully realized for people with SCI. Importantly, these findings do not imply that all people living with SCI in Sweden have a bad life, but rather aim to highlight the potential for improvement.

Significant medical advancements evolving rehabilitative focus over the past decades have vastly increased the possibilities for achieving a satisfactory quality of life with SCI (1–3). The literature review performed in study [b] revealed many research questions that have been addressed with emerging evidence, but not fully answered (listed in Appendix 1 in study [b]), indicating that researchers are currently investigating the questions important to people living with SCI. Nevertheless, there remains a need for further studies. Moreover, implementing the results is partly a matter of financial resources. The surrounding systems must provide the best possible care and rehabilitation, which often means combining different interventions and being flexible regarding individual contexts despite limited resources, to enhance quality of life for people with SCI. While this study highlights the needs, how policymakers and healthcare professionals make prioritizations goes beyond the scope of the Swedish needs assessment project.

For many individuals living with a disability, such as SCI, means having a stronger dependency on laws and regulations. Unfortunately, laws and regulations may not always be sufficiently flexible and are often hard to change. Our results highlight the need for laws and regulations to acknowledge the complexity and individual needs of persons living with SCI – throughout the life span. We hope that our results can guide government and authorities to better support human rights and, consequently, to enhance life for people with SCI. Importantly, knowledge translation, especially among authorities and decision-makers, is essential to achieve a better general understanding of the implications and complexity of SCI in everyday life. Consequently, an increased understanding of what it means to live with SCI can improve the attitudes of the authorities, prompting them to better support the individual’s responsibility and control over one’s life.

Throughout the project, the importance of family and close ones for an enhanced life was evident. Sustaining an SCI imposes a heavy burden on and forces major changes for all relations. Notably, the divorce rate is higher if 1 partner sustains an SCI, compared to the general population (14). Support for family and close ones is rarely a high priority in the initial rehabilitation, particularly with a shortened length of stay (15); however, our results emphasize that supporting relatives is important for achieving an enhanced life with SCI.

In conclusion, the Swedish needs assessment project results demonstrate that most people living with SCI *want to be experts* on their own injury and its unique consequences, to be in control and to ensure an enhanced life. However, the complexity of living with SCI requires engagement with specialist care from time to time throughout the lifespan. To some extent, people living with SCI also *need to become experts* due to the lack of knowledge about SCI in non-specialized health care and in society. To be able to become an expert, it is essential that they are provided the necessary knowledge and support. Further increased knowledge, understanding, and flexibility from authorities, society, and non-specialized healthcare providers, together with positive attitudes, are essential for persons with SCI to have an enhanced life. Importantly, this must continue throughout the lifespan, because new needs will emerge in association with both the long-term consequences of SCI and “normal” everyday life changes. The findings further reveal a need for better engagement with family members, peer supporters, and other actors in the individual’s immediate environment, and for these people to be viewed as resources to promote and support the willingness to take responsibility and control over one’s life.

Overall, the new evidence from the Swedish needs assessment project can guide researchers, healthcare providers, and knowledge translators with up-to-date information regarding the issues that matter most for improving the lives of people living with SCI. As the project was conducted within the Swedish context, caution is warranted when translating the findings to other settings, particularly those with different healthcare systems or levels of socio-economic development. Contextual factors may influence both the relevance and applicability of the results. Furthermore, the challenges of implementing new research in the complex SCI life puzzle highlight the importance of structures for implementing current and future research.

## Data Availability

All data from the project are available at the OFS platform: https://osf.io/73apw/
